# Transcriptomic analysis of lung development in wildtype and CFTR^*−/−*^ sheep suggests an early inflammatory signature in the CF distal lung

**DOI:** 10.1007/s10142-023-01050-y

**Published:** 2023-04-22

**Authors:** Jenny L. Kerschner, Alekh Paranjapye, Makayla Schacht, Frederick Meckler, Felix Huang, Gurkan Bebek, Arnaud J. Van Wettere, Misha Regouski, Iuri Viotti Perisse, Kenneth L. White, Irina A. Polejaeva, Shih-Hsing Leir, Ann Harris

**Affiliations:** 1 Department of Genetics and Genome Sciences, Case Western Reserve University School of Medicine, Cleveland, OH USA; 2 Department of Genetics, University of Pennsylvania, Philadelphia, PA USA; 3 Center for Proteomics and Bioinformatics, Cleveland, OH USA; 4 Department of Nutrition, Case Western Reserve University School of Medicine, Cleveland, OH USA; 5 Department of Animal, Dairy and Veterinary Sciences, Utah State University, Logan, UH USA

**Keywords:** Lung development, Transcriptomics, Cystic fibrosis, Inflammatory response, CF sheep

## Abstract

The precise molecular events initiating human lung disease are often poorly characterized. Investigating prenatal events that may underlie lung disease in later life is challenging in man, but insights from the well-characterized sheep model of lung development are valuable. Here, we determine the transcriptomic signature of lung development in wild-type sheep (WT) and use a sheep model of cystic fibrosis (CF) to characterize disease associated changes in gene expression through the pseudoglandular, canalicular, saccular, and alveolar stages of lung growth and differentiation. Using gene ontology process enrichment analysis of differentially expressed genes at each developmental time point, we define changes in biological processes (BP) in proximal and distal lung from WT or CF animals. We also compare divergent BP in WT and CF animals at each time point. Next, we establish the developmental profile of key genes encoding components of ion transport and innate immunity that are pivotal in CF lung disease and validate transcriptomic data by RT-qPCR. Consistent with the known pro-inflammatory phenotype of the CF lung after birth, we observe upregulation of inflammatory response processes in the CF sheep distal lung during the saccular stage of prenatal development. These data suggest early commencement of therapeutic regimens may be beneficial.

**Supplementary Information:**

The online version contains supplementary material available at 10.1007/s10142-023-01050-y.

## Introduction

The biological processes involved in human lung development are critical to establishing a healthy organ at birth and molecular and cellular events that perturb this normal differentiation may underlie respiratory disease. Though most aspects of the development of the mouse lung are very well studied (reviewed in (Basil and Morrisey 2020)), knowledge of human lung development remains limited by availability of biological material, and data are often derived from a limited number of time points (Czerwinski et al. 2020). However, some adult lung diseases clearly have embryonic or childhood origins (Nikolic et al. 2018). For example, studies on the human lung suggest that narrower airway lumens, which are also associated with genetic variants in the fibroblast growth factor 10 (*FGF10*) gene, are a susceptibility factor for chronic obstructive pulmonary disease (Smith et al. 2018). Another lung disease which may have a prenatal origin is cystic fibrosis (CF). Though the CF lung is apparently anatomically and physiologically normal at birth, the propensity for recurrent lung infections from the neonatal period onwards remains largely unexplained. Earlier work using human fetal tissues suggested that the human CF airways are in a pre-inflammatory state prior to the first lung infection (Tirouvanziam et al. 2000). Mid trimester human tracheal tissues were grafted into severe combined immunodeficient (SCID) mice where CF tissues exhibited a significant inflammatory imbalance when compared to non-CF samples, with elevated intraluminal interleukin 8 (IL-8) levels. Furthermore, direct observation of the immune cell population in CF and non-CF human fetal lungs identified an increased number of mast cells and macrophages through development in the CF tissues (Hubeau et al. 2001). More recently questions relating to CF lung development have been addressed in animal models of the disease. In the CF pig a defect in lung branching morphogenesis was suggested to underlie CF proximal airway disease (Meyerholz et al. 2018), though equivalent phenotypes were not observed in the CF sheep (Van Wettere et al. 2022). In CF ferrets, which do not exhibit an in utero lung phenotype, prior infection was not necessary for a muco-inflammatory lung phenotype to develop in the neonatal period (Rosen et al. 2018). Hence, there is still uncertainty about the timing and molecular events that predispose the CF airway to postnatal pathology, although there is limited opportunity to address these questions in humans. Here, we use a sheep model of CF (*CFTR*^*−/−*^) (Fan et al. 2018) and age-matched wild-type (WT) sheep of the same breed (American Romney) to perform a detailed transcriptional analysis through lung development. Historically, the sheep lung provided invaluable information of direct relevance to human lung development, biology, and physiology (reviewed in (Thorburn and Harding 1994)) (Dawes et al. 1953). We collected proximal and distal lung tissue separately at 6 time points through gestation, from which RNA was extracted and subjected to RNA-seq analysis. Detailed bioinformatic analysis revealed genes and biological processes that were differentially expressed between time points in WT and *CFTR*^*−/−*^ animals separately, and between WT and *CFTR*^*−/−*^ animals at each timepoint. The expression levels of key genes of interest were further validated by RT-qPCR. These data provide a detailed molecular profile of WT and *CFTR*^*−/−*^ lung development and differentiation, and also identify a proinflammatory phenotype before birth in the *CFTR*^*−/−*^ sheep distal lung.

## Materials and methods

### Animals

American Romney breed of domestic sheep (*Ovis aries*) were used in this study. All animal studies were approved and monitored by the Institutional Animal Care and Use Committee (IACUC) at Utah State University (IACUC protocol # 10089) and conformed to the National Institute of Health guidelines. WT sheep were bred according to standard protocols. Briefly, ewes were synchronized at estrus using an intramuscular (IM) injection of 2 mL EstruMate containing 250 μg/ml of cloprostenol, a synthetic analogue of Prostaglandin F2α (Merck Animal Health) and introduced to the ram on the same day. Ultrasonography around 40 days confirmed pregnancy status and day 1 of gestation was defined as ~ 48 h post injection. *CFTR*^*−/−*^ sheep were generated by somatic cell nuclear transfer (SCNT) using genetically modified sheep fetal fibroblast (SFFs) (CF 60 male, *CFTR* exon 2 targeted) derived from American Romney fetuses as described previously (Fan et al. 2018). SCNT recipient ewes were synchronized prior to embryo transfer as described elsewhere (Yang et al. 2016) with the following modification: 2.5 mL of EstruMate was given IM at the time of SYNCRITE Vaginal Sponges removal. A minimum of two fetuses were recovered at days 50, 65, 80, 100, and 120 of gestation and 147–150 days (term) for histological and molecular analysis as described below. WT term animals were born naturally and time after birth until euthanasia was recorded. The number of fetuses analyzed was limited by the stochastic frequency of singleton, twin, and triplet pregnancies, the need to collect all animals in a single breeding season to minimize natural variation, and the high cost of large animal work. However, since all animals were inbred American Romney sheep we expect natural variation to be less than in human tissues.

### Histopathologic analysis

A necropsy was performed on all fetuses collected, to examine for gross lesions and the findings were documented as described previously (Van Wettere et al. 2022). Proximal and distal lung tissue samples (defined in Fig S1A) were collected and fixed in 10% neutral buffered formalin for histology. Formalin-fixed tissue sections were processed and embedded in paraffin according to routine histologic techniques. Sections, 5 µm thick, were stained with hematoxylin and eosin (H&E), alcian blue, or periodic acid–Schiff (PAS) stain according to standard methods and examined by light microscopy.

### Tissue collection for RNA processing

Simultaneously with tissue collection for histology, proximal and distal lung tissue samples were snap frozen in liquid nitrogen and stored at − 80 °C for RNA purification. Tissues were homogenized in TRIzol (Thermo Fisher, 15596018) in a dry ice-cooled MP Biomedicals FastPrep-24 Classic homogenizer using Lysing Matrix D tubes (MP Biomedicals, 116913100) for 1 or 2 × 20 s cycles at 4.0 m/s. RNA was isolated using the TRIzol manufacturer’s protocol. RNA quality was assessed by NanoDrop, formaldehyde gel electrophoresis, and Tapestation prior to choosing high quality samples for RNA-seq.

### RNA sequencing and data analysis

RNA samples passing strict QC criteria were used as templates for cDNA library synthesis with oligo dT priming, and amplified libraries were pooled and sequenced (100 bp paired ends) on a NovaSeq 6000 machine. Raw sequence reads were aligned to the Oar_v4.0 (oviAri4) genome of the Texel sheep breed with STAR version 2.7.0e_0320 (https://github.com/alexdobin/STAR) (Dobin et al. 2013). Aligned reads were assigned to genomic features with featureCounts version 1.6.3 in the Subread package (http://subread.sourceforge.net/) (Liao et al. 2014). Gene expression analysis to identify differentially expressed genes (DEGs) was performed using edgeR version 3.34.1 (Chen et al. 2016). Genes were filtered by the default minimum count defined by library sizes and then normalized by tagwise dispersion. DEGs were filtered for those with a > 1.5 or ≤ 1.5 fold change (log_2_ ± 0.58) and an adjusted *p*-value ≤ 0.001 (Tables S1, S2 and S3). Further evaluation with an adjusted *p*-value ≤ 0.01 increased the number of DEGs as expected but did not substantially alter the biological processes identified in the subsequent gene ontology analysis (data not shown). Data are deposited at GEO:GSE202019.

### Gene ontology analyses

DEGs were filtered to enrich for genes with a fold change ≥ 1.5 and negative binomial adjusted *p*-value ≤ 0.001. RNA-seq gene lists were read into the gProfiler (version: e107_eg54_p17_bf42210, for individual WT and *CFTR*^*−/−*^ time courses; e106_eg53_p16_65fcd97, for WT vs *CFTR*^*−/−*^ at each time point) (Reimand et al. 2016) or DAVID gene ontology program and database (v2022q4 for individual WT and *CFTR*^*−/−*^ time courses; v2022q2 for WT vs *CFTR*^*−/−*^ at each time point) (Huang da et al. 2009; Raudvere et al. 2019; Sherman et al. 2022). gProfiler was run using default parameters, after selecting “Ovis aries (Sheep) (texel)) (version Oar_v3.1) annotated genome with Benjamini–Hochberg correction for multiple testing. Of note, the updated Oarv4.0 is not incorporated into the database.

### Reverse transcriptase quantitative PCR

Total RNA was extracted as described above and cDNA prepared using Taqman Reverse Transcription Reagents (Thermo Fisher, N8080234) with random hexamers, or oligo dT for *ANO1*, *KNG1*, *CHI3L1*, and *C4A* only. Using primers listed in Table S4 (all validated for primer efficiency), qPCR was performed with SYBR Green PCR Master Mix (Thermo Fisher, 4368708). Target gene expression was normalized to the mean of 2 control genes: ovine succinate dehydrogenase complex flavoprotein subunit A (*SDHA*) and glucose 6 phosphate dehydrogenase (*G6PD*) (Vorachek et al. 2013).

## Results

### The developmental transcriptome of WT and *CFTR*^*−/−*^ sheep proximal and distal lung tissue

We showed previously that proximal and distal lung tissue from *CFTR*^*−/−*^ animals did not show gross histological changes from WT animals through gestation (Van Wettere et al. 2022). To look for more subtle changes in lung biology that might not be evident on histology, we performed bulk RNA-seq on lung tissues from 6 time points through gestation. Tissues were taken from proximal and distal lung (regions defined in Fig. S1A) from WT and *CFTR*^*−/−*^ animals (2 of each) at 50-, 65-, 80-, 100-, and 120-day animals during gestation and 147-day (term) (Fig. S1B).

Principal component analysis (PCA) of RNA-seq data is shown in Fig. S2A, B, where samples are largely seen to cluster by developmental time point and genotype. The predominant principal component was defined by the progression along the timecourse. In the proximal CF lung samples replicates at day 65 and day 80 showed more variation and divergence from the same time points in the WT lung tissues, which were very similar to each other (Fig. S2A). Comparing global transcriptomic changes between time points in proximal lung, the 120-day to term interval showed the greatest changes, particularly in WT animals. Among distal lung tissue samples there was close clustering by time point and relatively little variation between time or genotype at 65 and 80 days (Fig. S2B). The major divergence in distal lung transcriptomes occurred between 100 and 120 days and between 120 days and term, irrespective of genotype.

### Differentially expressed genes through gestation in WT sheep proximal and distal lung

First, we conducted pairwise comparisons of differentially expressed genes (DEGs) between each time point of gestation in WT sheep lungs (Table S1). DEGs were identified using a stringent *p*-value filter of ≤ 0.001 and log_2_fold change of ± 0.58 (± 1.5 fold change). We compared 65 days to 80 days, 80 days to 100 days, 100 days to 120 days, and 120 days to term. Proximal and distal lung samples were evaluated separately. For each sample set gene ontology process enrichment analysis was performed on the DEGs to identify upregulated and downregulated processes across gestation.

#### Proximal lung

**Fig. 1:**
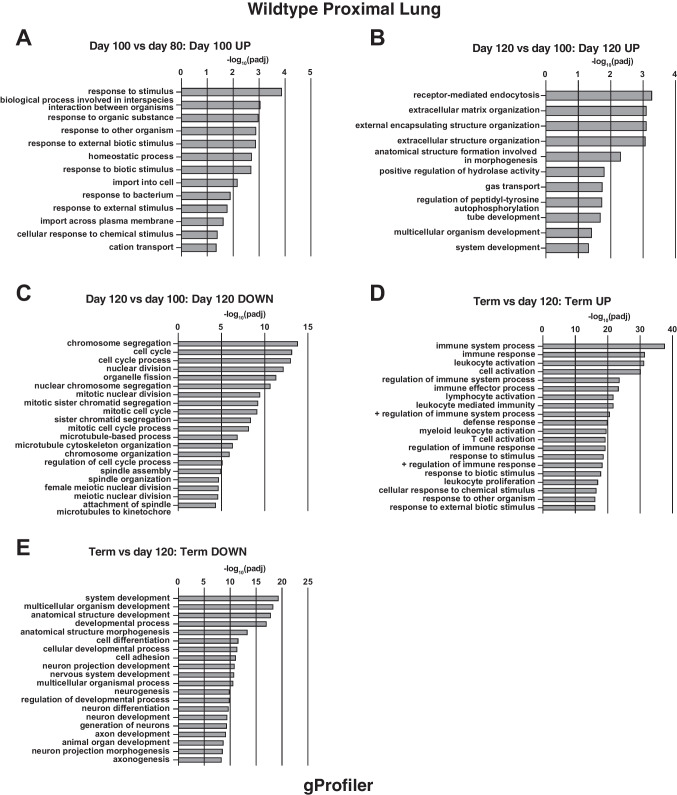
Gene ontology process enrichment analyses of differentially expressed genes between gestational time points in WT sheep proximal lung. Differentially expressed genes were filtered to enrich for genes with a fold change ≥  ± 1.5 and Benjamini–Hochberg adjusted *p*-value ≤ 0.001. Gene ontology analysis by gProfiler and the top 20 biological processes (BP) are shown. **A** BP from genes upregulated at 100 compared to 80 days; **B** BP from genes upregulated at 120 compared to 100 days; **C** BP from genes downregulated at 120 compared to 100 days; **D** BP from genes upregulated at term compared to 120 days; **E** BP from genes downregulated at term compared to 120 days

At the earlier stages of development before 100 days, few gene ontology terms were annotated between time points. Comparing 100 and 80 days, upregulation of multiple genes involved in response to external biological stimuli are annotated in gProfiler (Fig. 1A). Also, increased expression of genes associated with cell adhesion involving integrins and fibrillar collagens are identified in the DAVID analysis (Fig. S3A). Comparing 120 and 100 days, there are many more significantly upregulated genes and gProfiler annotates those involved in extracellular matrix, anatomical structures and importantly, gas transport (Fig. 1B). Concurrently, between these two time points, there is a substantial downregulation of genes involved in the cell cycle and cell division (gProfiler Fig. 1C, DAVID Fig. S3B). Next, comparing 147 days (term) to 120 days, among the most significantly upregulated genes are those encoding multiple components of the adaptive immune response, encompassing many genes with different roles in immune function (gProfiler, Fig. 1D). Some of the same upregulated genes are identified in the inflammatory response in addition to the immune response annotated by DAVID analysis in the same term to 120-day comparison (Figure S3C). There are also a substantial number of downregulated genes between 120 days and term that have roles in development and morphogenesis (gProfiler, Fig. 1E, DAVID, Fig. S3D).

#### Distal lung

**Fig. 2:**
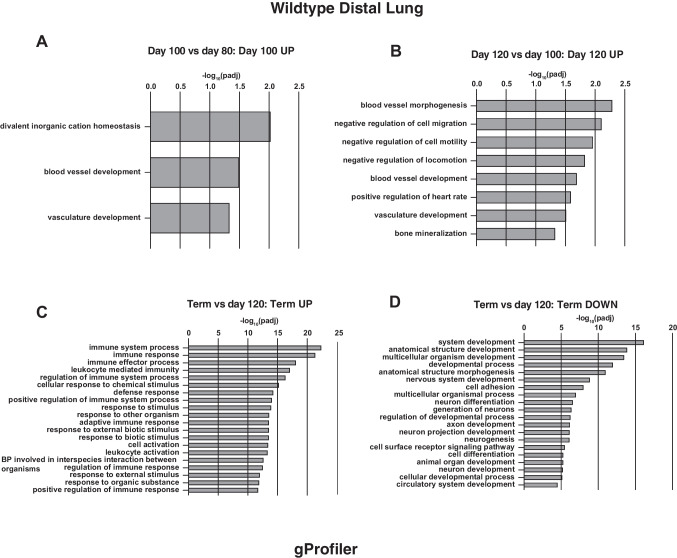
Gene ontology process enrichment analyses of differentially expressed genes between gestational time points in WT sheep distal lung. Analysis methods as described in Fig. 1. **A** BP from genes upregulated at 100 compared to 80 days; **B** BP from genes upregulated at 120 compared to 100 days; **C** BP from genes upregulated at term compared to 120 days; **D** BP from genes downregulated at term compared to 120 days

Next, comparing developmental time points in the distal lung, again there are few significant processes identified between 65 and 80 days of gestation, although between 80 and 100 days, upregulation of several solute carrier family member genes identifies solute:cation/sodium symporter activity as significant molecular functions and also the divalent inorganic cation homeostasis biological processes, albeit with a modest p-value (Fig. 2A). Comparing 120- and 100-day gestation, gProfiler identified negative regulation of cell motility and migration, and significant upregulation of genes involved in blood vessel morphogenesis processes, although again these are driven by a small number of genes and a statistically significant though marginal *p*-value (Fig. 2B). As seen in the proximal lung, more substantial changes in gene expression are seen between 120 days and term (Table S1). Multiple genes with a role in immune response processes are upregulated during this time interval, including both the innate and adaptive immune responses, also the inflammatory response (gProfiler, Fig. 2C, DAVID, Fig. S4). Among downregulated genes are many involved in developmental and morphogenetic processes (gProfiler, Fig. 2D).

### Differentially expressed genes through gestation in *CFTR*^*−/−*^ sheep proximal and distal lung

Next, we conducted equivalent pairwise comparisons of DEGs between each time point of gestation in *CFTR*^*−/−*^ sheep lungs using the same filters of *p*-value ≤ 0.001 and log_2_fold change of ± 0.58 (± 1.5 fold change) (Table S2). Again, proximal and distal lung samples were evaluated separately, and we compared 65 days to 80 days, 80 days to 100 days, 100 days to 120 days, and 120 days to term. For each sample set, gene ontology process enrichment analysis was performed on the DEGs to identify upregulated and downregulated processes across gestation.

#### Proximal lung

**Fig. 3:**
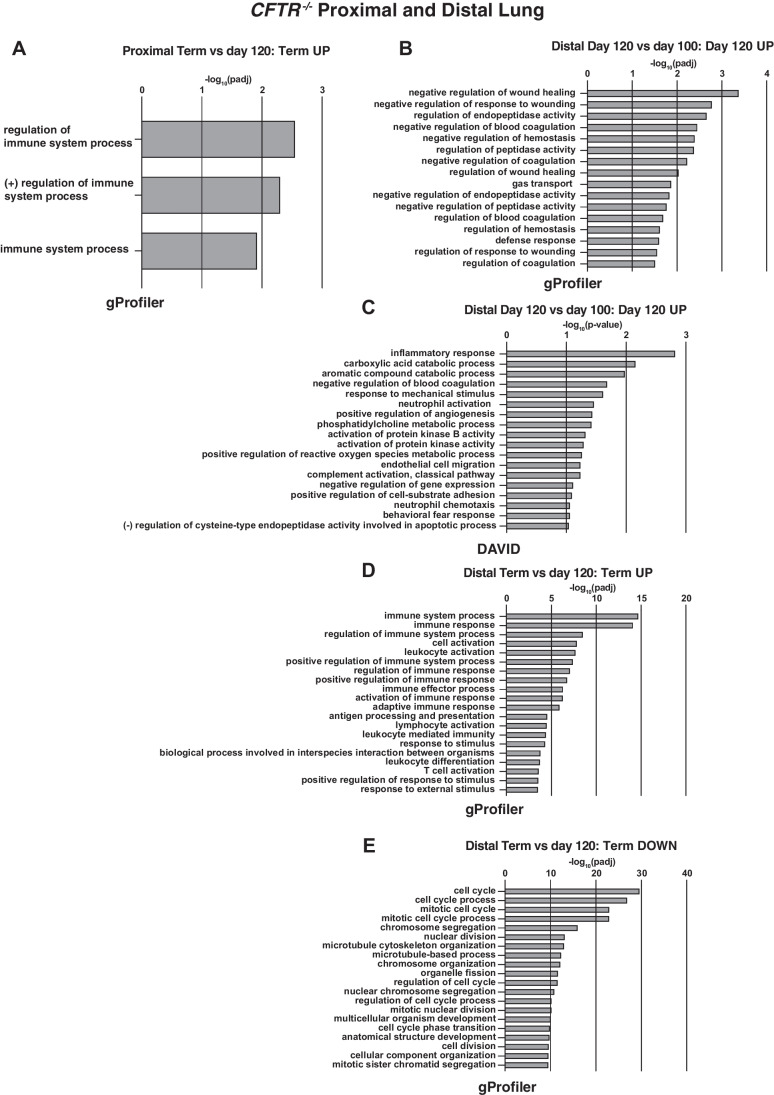
Gene ontology process enrichment analyses of differentially expressed genes between gestational time points in *CFTR*^*−/−*^ sheep proximal and distal lung. Analysis methods as described in Fig. 1. **A** BP from genes upregulated at 120- compared to 100 days in proximal lung; **B** BP from genes upregulated at 120 compared to 100 days in distal lung by gProfiler; **C** BP from genes upregulated at 120 compared to 100 days in distal lung by DAVID; **D** BP from genes upregulated at term compared to 120 days in distal lung by gProfiler; **E** BP from genes downregulated at term compared to 120 days in distal lung by gProfiler

As for the WT animals, before 100 days, few gene ontology processes were identified between time points, perhaps in part due to the divergence of individual replicates (Fig. S2A). However, some DEGs of specific biological interest in lung function were identified and will be discussed later. Comparing 100- to 80- day gestation, as for WT proximal lung at the same time points, cation symporter is a molecular function identified by both gProfiler and DAVID, although apart from *SLC1A1*, the upregulated solute carrier genes are different in WT and CF animals. Contrasting 120- to 100-day gestation, gene ontology molecular functions annotated from upregulated genes were divergent in *CFTR*^*−/−*^ animals from those seen in WT animals. In the *CFTR*^*−/−*^ animals, oxidoreductase and aldehyde oxidase molecular functions were identified, albeit driven by very few genes (including aldehyde oxidase 1, *AOX1*). Also of interest was the upregulation of genes involved in the innate immune response and anti-inflammatory response, which are included in the cellular compartment defined as extracellular space. These genes include surfactant proteins (*SFTPA1*, which is also upregulated in WT lung at this stage, and *SFTPD*), secretoglobins (*SCGB1A1*), and the PLUNC/BPI fold containing family B member 1 (*BPIFB1*). However, prior to 120 days, no significant gene ontology biological processes are called, only molecular functions. Lastly, comparing term to 120 days in *CFTR*^*−/−*^ animals, the most upregulated gene (log_2_fold change > 9) is the macrophage receptor with collagenous structure (*MARCO*), a key component in innate antimicrobial immunity. However, there are many fewer DEGs between these two time points in the CF animals (~ 150) compared to WT (~ 1000). Regulation of immune system is called as a significant process by gProfiler when comparing these timepoints (Fig. 3A), although few processes are called by DAVID.

#### Distal lung

First comparing *CFTR*^*−/−*^ distal lung between 80 and 65 days, only ~ 20 genes met the stringent filter of ± 1.5 fold change (log_2_ ± 0.58) and an adjusted *p*-value ≤ 0.001 and so gene ontology analysis was not fruitful. However, robust upregulation of both *SFTPB* and *SFTPD* was of significant interest (Table S2). Comparing 100 and 80 days, only ~ 30 genes passed the filter and these again called the cation symporter molecular function. The comparison of 120 and 100 days was more fruitful with ~ 140 DEGs, and from the upregulated genes, gProfiler annotated negative regulation of response to wounding, negative regulation of endopeptidase activity and of coagulation, and also enhancement of both gas transport and defense response (Fig. 3B). Upregulated DEGs associated with the defense response included *CXCL8* (encoding the chemokine IL-8), kininogen (*KNG1*), chitinase 3-like 1 (*CHI3L1*), complement C4A (*LOC101123672*), and WAP four-disulphide core domain-1 (*WFDC1*). Of note, *WFDC2* is also robustly upregulated (Table S2). DAVID identifies the same gene set contributing to upregulation of the inflammatory response (excluding the *WFDC*s) (Fig. 3C). Between 120-day gestation and term, ~ 720 genes are differentially expressed and many of the upregulated genes are components of the adaptive immune response, with multiple associated processes identified by both gProfiler (Fig. 3D) and DAVID (Fig. S5A). These include activation of lymphoid lineage cells, regulation of immune system processes, and antigen processing and presentation. Among downregulated genes at term compared to 120 days are many involved in the cell cycle (gProfiler, Fig. 3E, DAVID Fig. S5B).

### Differentially expressed genes comparing WT and *CFTR*^*−/−*^ animals at each time point

Next, we compared the gene expression profiles of tissue samples from *CFTR*^*−/−*^ animals to WT animals at each time point (Table S3), identified DEGs with the same filters as used above and then performed gene ontology process enrichment analysis.

#### Proximal lung

Upregulated genes in the *CFTR*^*−/−*^ lungs compared to WT at 65 days contribute to multiple development processes, although *p*-values were generally low and so the data are not presented in figures herein. Among these, upregulated genes (Table S3) are insulin like growth factor binding protein 5 (*IGFBP5*) and myocardin (*MYOCD*) both of which regulate aspects of smooth muscle cell replication, differentiation, and migration, in addition to other functions. At 80 days, higher expression in *CFTR*^*−/−*^ lungs than WT of scavenger receptor cysteine-rich family member with 5 domains (*SSC5D*), extracellular matrix protein 1 (*ECM1*), and integrin subunit alpha 7 (*ITGA7*) predicts extracellular matrix (ECM) binding as a molecular function in gProfiler. SSC5D is active in the ECM where it has multiple functions in laminin and fibronectin binding in addition to scavenger receptor activity (Miro-Julia et al. 2011). It has an important role in defense response since it binds to pathogen-associated molecular patterns (PAMs) on the cell walls of bacteria and fungi (Bessa Pereira et al. 2016). Also, at 80 days, a significant reduction in expression of the chemokines CXCL10 and CXCL11 is observed in *CFTR*^*−/−*^ lungs, although in the absence of differential expression of other chemokines, the importance of this observation is unclear. The ~ 80 and ~ 140 DEGs between *CFTR*^*−/−*^ and WT lungs at 100 and 120 days respectively did not identify any significant processes in either gProfiler or DAVID. However, it was notable that secretoglobin family 1A member 1 (*SCGB1A1*) was among the most differentially upregulated genes in *CFTR*^*−/−*^ compared to WT proximal lungs at 120 days. At term, the ~ 400 DEGs between *CFTR*^*−/−*^ and WT predict multiple highly significant biological processes, with anatomical structure development/ morphogenesis having the highest *p*-values, but response to hypoxia and related processes also reaching statistical significance. Among upregulated genes driving the hypoxia signature are Egl-9 family hypoxia inducible factor 3 (*EGLN3*), a cellular oxygen sensor that hydroxylates proline residues in hypoxia inducible factor 1 subunit alpha (HIF1A) and other targets (Kaelin and Ratcliffe 2008). Upregulation of hypoxia-related genes was identified in both *CFTR*^*−/−*^ animals analyzed at term, although more dramatically in one of them (Table S3). Cell cycle/cell division and microtubule cytoskeleton organization were among annotated processes from the downregulated genes in the *CFTR*^*−/−*^ animals compared to WT at term.

#### Distal lung

The ~ 250 DEGs between *CFTR*^*−/−*^ and WT animals at 65 days gestation did not identify significant processes of interest by gProfiler or DAVID. Similarly at 80 days gestation, although potassium channel activity was identified from upregulated genes in the *CFTR*^*−/−*^ animals by both enrichment analysis tools, the *p*-values were low and were associated with small (though statistically significant) changes in expression of a few genes including potassium voltage-gated channel subfamily members (*KCNH2* and *KCND3*). In contrast by 100 days gestation, the ~ 230 DEGs between *CFTR*^*−/−*^ and WT animals resulted in annotation of ion transport processes by gProfiler based on the significantly upregulated genes in the *CFTR*^*−/−*^ animals. Multiple genes contributing to these processes include the voltage-gated calcium channel cation channel sperm associated 4 (*CATSPER4)*, anoctamin 9 (*ANO9*) a member of the calcium-activated chloride channel which may not transport chloride, the ATP binding cassette transporter family members *ABCC5* and ABCC*10*, and polycystin 1 (*PKD1*). At 120-day gestation, the ~ 210 DEGs did not identify any robust signatures for biological processes between the genotypes. As for the proximal lung, at term, the ~ 640 DEGs predict anatomical structure development, angiogenesis, and response to hypoxia among the significantly upregulated genes annotated by gProfiler and downregulated genes are associated with cell cycle-related processes in the *CFTR*^*−/−*^ animals compared to WT. Many genes contribute to all these processes and indicate a term lung that is still undergoing development but shows reduced cell division in the term *CFTR*^*−/−*^ animals.

### Developmental expression of key genes relevant to lung epithelial differentiation

**Fig. 4:**
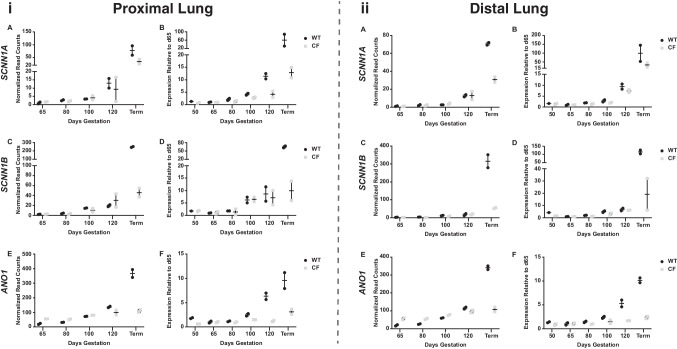
Developmental profiles of ion channel gene expression in WT and *CFTR*^*−/−*^ sheep proximal and distal lung. *SCNN1A*, *SCNN1B*, and *ANO1* expression is shown. Data on the left are expression profiles in proximal lung and on the right in distal lung. Normalized read counts from RNA-seq data in **A**, **C**, and **E**. RT-qPCR validation of expression in **B**, **D**, and **F** with values relative to expression at 65 days. WT samples are black dots and *CFTR*^*−/−*^ (CF) samples are grey squares. Values plotted are from 2 biological replicates at each time point for RNA-seq and the mean of technical replicates for each biological sample for RT-qPCR

**Fig. 5:**
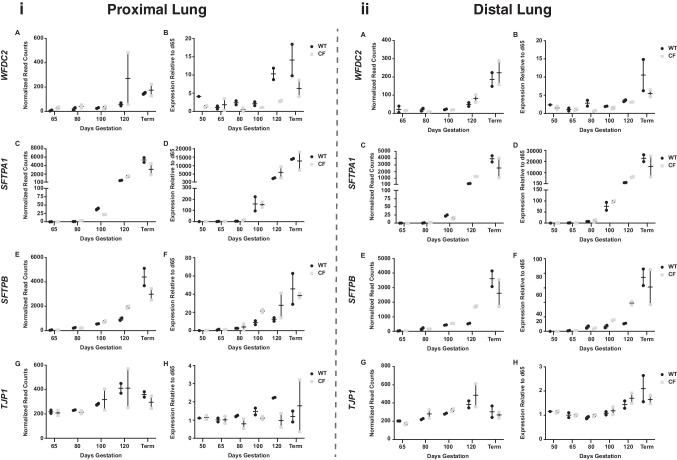
Developmental profiles of innate immune and surfactant gene expression in WT and *CFTR*^*−/−*^ sheep proximal and distal lung. *WFDC2*, *SFTPA1*, and *SFTPB* expression is shown, with *TJP1* as a control for minimal expression changes through gestation. Data on the left are expression profiles in proximal lung and on the right in distal lung. Normalized read counts from RNA-seq data in **A**, **C**, **E**, and **G**. RT-qPCR validation of expression in **B**, **D**, **F**, and **H** with values relative to expression at 65 days. WT samples are black dots and *CFTR*^*−/−*^ (CF) samples are grey squares. Values plotted are from 2 biological replicates at each time point for RNA-seq and the mean of technical replicates for each biological sample for RT-qPCR

We then took a targeted approach to the expression profiles of individual genes with a known role in lung development and relevance to lung physiology in CF. For each gene, normalized read counts from the RNA-seq data were graphed against developmental time point for *CFTR*^*−/−*^ and WT animals and then validated by RT-qPCR for both proximal and distal lungs (Figs. 4 and 5). We first considered ion channels: the *SCNN1A-* and *SCNN1B*-encoded subunits of the epithelial sodium channel (ENaC), (Fig. 4A–D), the calcium-activated chloride channel TMEM16A/anoctamin (*ANO1*) (Fig. 4E, F), and the solute carrier/anion exchanger *SLC26A9* which mediates chloride/bicarbonate exchange and chloride-independent bicarbonate release. Functional interactions of each of these channels with CFTR are well established (Knowles et al. 1983); (Stutts et al. 1995); (Bertog et al. 2000); (Caputo et al. 2008); (Benedetto et al. 2017). The expression of the small conductance chloride ion channel encoded by *CFTR* will be considered later. In both the proximal and distal lungs, *SCNN1A* and *SCNN1B* showed similar expression profiles with low levels at 50 and 65 days, then a gradual increase through 100 days, followed by a greater increase by 120 days, with the highest levels at term (Fig. 4A–D), consistent with the role of ENaC in the post-natal airway epithelium. Levels of the two subunits were similar at early time points in the WT and *CFTR*^*−/−*^ animals. However, later in gestation, *SCNN1A* expression in *CFTR*^*−/−*^ animals was noticeably lower by 120 days in the proximal lung, and in both proximal and distal lungs at term (Fig. 4A, B). At term, *SCNN1B* levels were also lower in both regions of the lung in *CFTR*^*−/−*^ animals (Fig. 4C, D). *ANO1* expression increased gradually through gestation, but a ~ threefold increase in normalized read counts between 120 days and term was only seen in WT animals, with expression levels showing little change over this period in the *CFTR*^*−/−*^ animals (Fig. 4E, F). In the data shown in Fig. 4, all forms of the *ANO1* transcript were amplified in the qRT-PCR reaction; however, we noted that an isoform-restricted primer set showed a different expression pattern, indicating temporal alternative splicing of the gene (Ferrera et al. 2009) (data not shown). *SLC26A9* expression was rather variable between animals of the same genotype and gestational age making it difficult to interpret expression profiles. However, the normalized read counts from RNA-seq suggested that *SLC26A9* expression was higher in both proximal and distal lungs of *CFTR*^*−/−*^ animals compared to WT at all gestational ages in both regions of the lung (Fig. S6), consistent with its role in chloride/bicarbonate exchange and chloride-independent bicarbonate secretion (Ousingsawat et al. 2022).

Also, highly relevant to lung functions are the components of the innate immune response, which are challenging to study in the fetus prior to birth (Feyaerts et al. 2022). We examined two genes that are representative of these processes: WAP four-disulphide core domain 2 (*WFDC2*), the lung epithelial cell protease inhibitor that has a predicted role in lung surface liquid and innate immunity (Bingle et al. 2006), and the antimicrobial/defense response protein BPI fold containing family A member 1 (*BPIFA1*) (Bingle and Craven 2002). In both the proximal and distal lungs for both WT and *CFTR*^*−/−*^ animals, *WFDC2* is expressed at low abundance prior to 120-day gestation when it increases through to term (Fig. 5A, B). There is no consistent difference in expression levels between the genotypes. In both the proximal and distal lungs, *BPIFA1* expression was undetectable at most time points before birth in both genotypes (data not shown).

Lastly, we assayed the genes encoding surfactant proteins/secretoglobins (*SFTPA*1, *SFTPB*, and *SCGB3A2*) (Fig. 5C–F). *SFTPA*1 was expressed at low abundance prior to 80 days in the proximal lung of both genotypes, but by 100 days an ~ 100-fold increase in transcript abundance compared to 65 days was seen in both genotypes, which further increased to more than 1000-fold by 120 days before rising more moderately to term. In contrast, *SFTPB* levels rose more gradually through to term in both WT and *CFTR*^*−/−*^ animals (Table S1). In the distal lung, the profile of *SFTPA1* expression was very similar to that seen in the proximal lung but *SFTPB* expression increased more gradually through gestation in animals of both genotypes, with a large increase close to term. The abundance of *SCGB3A2* transcripts in proximal and distal lung increased > 200- and > 20-fold respectively, between 65 and 120 days of gestation, with higher levels at term particularly in the distal lung, but variation between individuals at each time point made these data difficult to interpret (data not shown). For comparison in these studies, we assayed the expression of a lung epithelial integrity protein (tight junction protein 1, *TJP1*), which as expected did not alter significantly through gestation in proximal or distal lung irrespective of genotype (Fig. 5G, H).

### Expression of the cystic fibrosis transmembrane conductance regulator gene through gestation

**Fig. 6:**
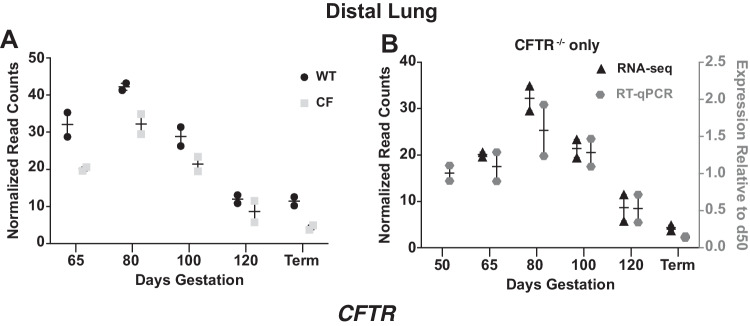
Developmental profile of *CFTR* gene expression in WT and *CFTR*^*−/−*^ sheep distal lung. **A**. Normalized read counts from RNA-seq data show *CFTR* expression through gestation in WT and *CFTR*^*−/−*^ (CF) sheep distal lung: **B**. Validation of RNA-seq data by RT-qPCR for *CFTR*^*−/−*^ animals only

Of particular interest, in the light of our earlier work on the developmental expression of the cystic fibrosis transmembrane conductance regulator (*CFTR*)(Broackes-Carter et al. 2002), in which the transcript was assayed solely by RT-qPCR, was the expression profile of the gene in the RNA-seq data from WT and *CFTR *^*−/−*^ animals. We observed that the profiles of normalized read counts were similar between proximal and distal lung in both WT and *CFTR*^*−/−*^ animals, with highest expression levels at 80-day gestation followed by a rapid decline across 100 and 120 days at which point levels were similar to those in the term lung (Fig. 6). These data closely replicate our earlier findings in WT animals of a different sheep breed (Broackes-Carter et al. 2002). Of note, the abundance of *CFTR* transcripts assayed by RNA-seq in the proximal and distal lung of *CFTR*^*−/−*^ animals was consistently lower than in the WT animals (Fig. 6A), possibly due to nonsense-mediated decay (NMD) of the *CFTR*^*−/−*^ transcript, which contains a frameshift mutation in exon 2 (Fan et al. 2018).

### Validation of differential upregulation of defense response/immune genes between 100 and 120 days in *CFTR*^*−/−*^ distal lung alone

**Fig. 7:**
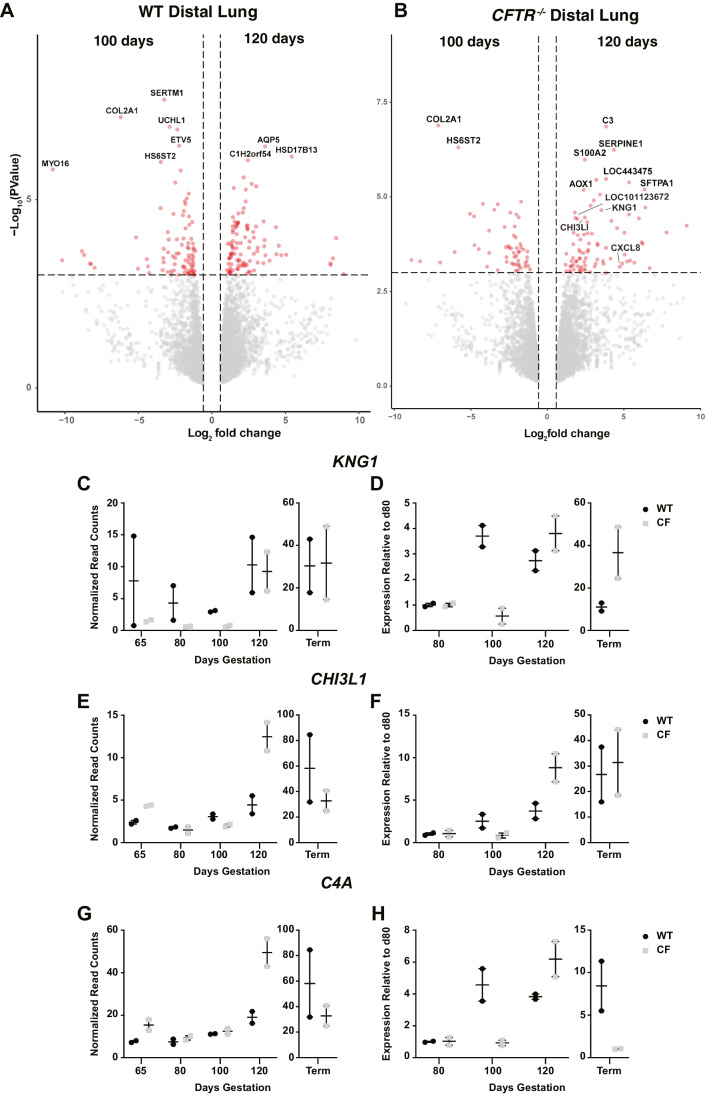
Enhanced relative expression of inflammatory response genes at 120 days of gestation compared to 100 days in distal lung from *CFTR*^*−/−*^ (CF) animals. **A**, **B** The volcano plots shows differential gene expression comparing RNA-seq data from 100 day and 120-day distal lung tissue from **A** WT or **B** *CFTR*^*−/−*^ animals. Differentially expressed genes were filtered to enrich for genes with a fold change ≥  ± 1.5 and *p*-value ≤ 0.001. *KNG1*, *CHI3L1*, *C4A (LOC101123672)*, and *CXCL8* are highlighted on **B**. **C**, **E**, **F** Normalized read counts for *KNG1*, *CHI3L1*, and *C4A* respectively. **D**, **F**, **H** RT-qPCR validation of the same genes with expression plotted relative to expression levels at 80 days

In the RNA-seq results presented above comparing 100 and 120 days, we noted that defense response and inflammatory response were annotated from the significantly upregulated genes by gProfiler and DAVID respectively in CF distal lung alone (Fig. 3B, C). Given the relevance of lung inflammation to the CF phenotype after birth (Schupp et al. 2020), we validated these results by RT-qPCR. Figure 7A and B show volcano plots with upregulated genes comparing 120—and 100-day distal lung tissue from WT and *CFTR*^*−/−*^ animals. Transcripts of *KNG1*, *CHI3L1*, *LOC101123672 *(*C4A*), *C3*, and *CXCL8* (encoding IL-8) at 120 days are marked in Fig. 7B and plotted through gestation in Fig. 7C, E, and G. To validate the RNA-seq data, we used RT-qPCR to measure the abundance of *KNG1*, *CHI3L1*, and *C4A* transcripts (Fig. 7D, F, H). Focusing on the relative increase in transcripts of each of these genes at 120 days compared to 100 days, all are elevated to a greater extent in the *CFTR*^*−/−*^ distal lung tissue than in WT distal lung at the same time points (Fig. 7C–H). *CXCL-8* transcript levels were of very low abundance and so were not analyzed further. Together these data suggest an early manifestation of an inflammatory phenotype in the CF lung at 120 days, prior to the major upregulation of immune response genes at term that occurs in both proximal and distal lungs of WT and *CFTR*^*−/−*^ animals (Fig. 1D, Fig. 2C, respectively).

## Discussion

The advances in functional genomics protocols that enable the type of investigation, we describe here were made many decades after restrictions on the use of human fetal tissues were enacted in many countries. As a result, the use of relevant large animal models of human disease can make a unique contribution to understanding developmental phenotypes. Although transcriptomic analysis that is based on bulk tissue RNA samples lacks the precision of single cell RNA-seq (scRNA-seq) and the detailed information on the differentiation and migration of individual cell types that it provides, it can still yield important insights into organ development and disease-associated deficits.

Human lung development is separated into 5 overlapping stages, largely based upon embryological transitions (reviewed in (Burri 1984) (Nikolic et al. 2018)), and by convention is measured in weeks post-conception (pcw). The stages are embryonic (4–7 pcw), pseudoglandular (5––17 pcw), canalicular (16–26 pcw), saccular (24–38 pcw), and alveolar 36 pcw to 3 years (Fig. S1B). During the embryonic stage, the bilateral lung buds arise from the foregut endoderm and between 4 and 5 weeks a rapid branching of the buds establishes the lobular structure of the lung. Branching morphogenesis continues through the pseudoglandular stage and cartilaginous airways develop, with epithelial branching and differentiation accompanied by blood vessel development. The canalicular phase is marked by growth of airways and additional epithelial branching to generate the future alveolar regions. Small blood vessels become closely associated with the distal airspaces, where alveolar epithelial differentiation commences. At the start of the saccular stage, the small airways terminate in thin-walled saccules and during this period the respiratory airspaces undergo major expansion, accompanied by a loss of interstitial tissues. Also during this time, the capillary network envelops the saccules and within these, ongoing alveolar epithelial differentiation is marked by the start of surfactant production. Note in our study transcriptional upregulation of surfactant protein gene expression commences during the canalicular stage (Fig. 5). Finally, during the alveolar stage, saccules become divided into alveoli as septa develop from the saccular walls, enabling the large increase in surface area that is required for gas exchange. Concurrently, capillary differentiation establishes the network required to support these surfaces. As mentioned earlier, the development of the sheep lung is very similar to that of the human lung (reviewed in (Thorburn and Harding 1994)) although the gestation period differs (human (usually defined as 280 days/40 weeks though may be shorter (Jukic et al. 2013)) and sheep (147 days)). We examined the lung histology of WT animals collected through the time course (Van Wettere et al. 2022) to broadly define the developmental stages at 50, 65, 80, 100, and 120 days and term (Fig. S1B). Of note, earlier work on sheep lung development based upon light and electron microscopic analysis of single fetuses at each time point defined only four non-overlapping stages (embryonic, pseudoglandular 40 to 80 days, canalicular 80 to 120 days and alveolar 120 to term) (Alcorn et al. 1981). In this manuscript, we follow the overlapping model and developmental stages used in analysis of the human and mouse lung (Burri 1984), (Nikolic et al. 2018) as they are more detailed, and reflect our histological observations. Since we define collection points by time rather than anatomical stage, this does not alter data interpretation.

There are two major aspects of interest in the work presented here, the first being the detailed transcriptomic analysis of WT lung development, which has not been done previously in a large animal, and the second is the differences observed in the *CFTR*^*−/−*^ animals. For WT proximal lung, our data show that upregulated genes are significantly involved in biological processes relating to response to external stimuli, both chemical and biological between 80 and 100 days, extracellular matrix organization and preparation for gas transport between 100 and 120 days, and most notably, the innate and adaptive immune responses at term, which is accompanied by marked downregulation of genes involved in developmental processes. In WT distal lung, upregulation of genes involved in cation homeostasis and blood vessel development is seen between 80 and 100 days, with the latter processes also annotated for genes upregulated between 100 and 120 days. The changes in biological processes between 120 days and term are the same as observed in proximal lung. For *CFTR*^*−/−*^ proximal lung, when using the stringent filters for statistical significance applied here few biological processes were identified until term when immune system processes were annotated from genes upregulated compared to 120 days of gestation. However, in the *CFTR*^*−/−*^ distal lung, gProfiler annotates wound healing, coagulation, and defense response from genes upregulated between 100 and 120 days and using the same gene list DAVID identifies the inflammatory response and neutrophil activation over this time period. As in WT lungs, there is a marked upregulation of genes involved in both innate and adaptive immune system processes between 120 days and term in the *CFTR*^*−/−*^ distal lung, which is accompanied by downregulation of cell cycle-associated genes rather than the developmental processes seen in WT. The mechanisms underlying this difference in downregulated genes at term in the *CFTR*^*−/−*^ and WT lung are unclear. Although it is possible that earlier changes in the *CFTR*^*−/−*^ lung have a differential effect on lung development, for example through inflammation and associated tissue remodeling (reviewed in (Regamey et al. 2011)), the mechanisms may also reflect differences between cloned and non-cloned animals. This topic, which is extensively discussed in the literature, is addressed further below. The recent availability of non-cloned *CFTR*^*−/−*^ sheep naturally bred from heterozygous (*CFTR*^*+/−*^) ewes and rams will enable us to address these processes in detail.

The relative lack of significant biological processes called at consecutive time points through development in the *CFTR*^*−/−*^ animals when compared to WT, particularly in the proximal lung, is somewhat surprising since we did not detect any consistent abnormalities in lung structure or development through gestation by histological analyses (Van Wettere et al. 2022). It is possible that individual variation between samples is greater in the *CFTR*^*−/−*^ proximal lung samples (see PCA plot in Fig. S2A), which would impact downstream bioinformatic analysis. However, it is not clear why this variability would specifically impact the *CFTR*^*−/−*^ animals, as we followed a clearly defined protocol for collection of proximal and distal lung samples of both genotypes (Fig. S1A). Despite this, it is possible that sample bias on a small number of samples could be a contributing factor, particularly in the proximal lung where the inclusion of a variable amount of epithelium from the larger airways could substantially alter the transcriptome.

Another aspect of this analysis that warrants consideration is the fact that the *CFTR*^*−/−*^ animals used here were cloned from somatic cells (Fan et al. 2018). Although there are no reports of cloning protocols having a specific effect on lung development, it is well established that deficits in epigenetic reprogramming may impact cloned animals (reviewed in (Simmet et al. 2020)). In our preliminary studies comparing *CFTR*^*−/−*^ animals generated by natural breeding or by cloning, we observed some statistically significant differences in the transcriptomes of term proximal and distal lung tissue. However, these changes relate more to general development processes rather than lung specific functions (data not shown). Another factor affecting the transcriptome of the term lungs that warrants consideration is the precise time of euthanasia and tissue collection after birth. As part of the alveolar stage of development, the lung epithelium undergoes a period of rapid change immediately after birth. Pathways of ion transport become modified to facilitate respiration in air and the alveolar surface is coated with the necessary balance of surfactant proteins and antimicrobial agents. Indeed, we confirmed the dramatic upregulation of transcripts encoding these compounds between 100-day gestation and term in both regions of the lung and in WT and *CFTR*^*−/−*^ animals (Fig. 5), with *SFTPB* upregulation being more marked at 120 days in the *CFTR*^*−/−*^ animals. Because of these term-associated changes, we paid particularly attention to the time between birth and euthanasia of animals in these experiments.

Lastly, we consider our observations on the elevated inflammatory response in the distal lung of *CFTR*^*−/−*^ animals at 120 days compared 100 days. Chitinase-like proteins such as CHI3L1 (YKL-40) are members of the glycoside hydrolase family 18 proteins. Their role in innate immune pathways and tissue injury/repair are well characterized (reviewed in (Sutherland 2018)). Kininogen (*KNG1*) may have several functions in inflammation since it encodes two different proteins by alternative splicing. One of these, high molecular weight kininogen (HMWK) is required for blood coagulation, but bradykinin, which is released from it also has antimicrobial peptide properties and is associated with lung inflammation (Hayashi et al. 1998); (Wildi et al. 2022). Although rather few genes drive the biological process, this is possibly because we lose a substantial number of sheep genes when running the gene ontology pipelines. Many genes are absent from *Ovis aries* genes in the Gene Ontology Resource (http://geneontology.org) from which gProfiler and DAVID retrieve data. For example, *C3*, the most differentially upregulated gene in the 120 day *CFTR*^*−/−*^ distal lung (Fig. 7B, Table S2) is not included in the inflammatory response process called in the sheep. If analyzed within the *Homo sapiens* genome, the *C3* gene would be associated with multiple biological process terms, including “GO:0006954:inflammatory response.” We have manually converted many sheep LOC into known human genes to get further insights into the functional aspects of sheep lung biology and look forward to the updating of the Gene Ontology Resource with additional species. We validated the transcriptomic data generated by RNA-seq by RT-qPCR and are confident that these early changes in inflammatory gene expression are robust. These observations are of considerable importance in the context of CF lung disease after birth since it is still not certain why the CF lung is susceptible to early infection and inflammation. Earlier work had suggested that the human fetal CF lung was in a pre-inflammatory state (Tirouvanziam et al. 2000) and might have an imbalance of immune cells (Hubeau et al. 2001), but these observations preceded the availability of large-scale transcriptomic analysis such as we have done here. The identification of complement components (*C3* and *C4A*) among the most significantly upregulated genes between 100 and 120 days only in the *CFTR*^*−/−*^ distal lung warrants further discussion. In fact, *C3* is also upregulated in the *CFTR*^*−/−*^ proximal lung at this time point, although not in WT proximal or distal lung. Complement has a key role in the innate immune response as its activation causes local inflammation, the clearing and direct killing of pathogens and the initiation of the adaptive immune response (Lubbers et al. 2017). Although soluble complement system components are mainly synthesized in the liver, other membrane-bound components may be synthesized in specific cell types. At this point, it is not clear whether the C3 and C4A upregulation observed in the *CFTR*^*−/−*^ distal lung is occurring in mast cells, macrophages, or other immune cells in the developing lung, or indeed in epithelial cells. Future single cell RNA-seq analysis will likely clarify the cellular origin of the elevated complement component production. Irrespective of the cellular origin, the finding of early inflammation in the CF distal lung may have a profound impact on the management of post-natal disease and may help focus therapeutic regimens. The fetal sheep is particularly well suited for developing in utero therapies whether these utilize pharmacological (McGillick et al. 2014, 2022) or gene therapy (Abi-Nader et al. 2012; David et al. 2011) approaches.

## Supplementary Information

Below is the link to the electronic supplementary material.

Supplementary file1 (PDF 1715 KB)

Supplementary file2 (XLSX 8206 KB)

Supplementary file3 (XLSX 8754 KB)

Supplementary file4 (XLSX 355 KB)

Supplementary file5 (XLSX 10 KB)

## Data Availability

All transcriptomics data are available at GEO:GSE202019.
