# Supplementary material for: Transcriptomic analysis of lung development in wildtype and CFTR−/− sheep suggests an early inflammatory signature in the CF distal lung
Source: Funct Integr Genomics. 2023 Apr 22;23(2):135. doi: 10.1007/s10142-023-01050-y (PMC10121546; doi:10.1007/s10142-023-01050-y)
Supplement: Supplementary file 1 — Supplementary file1 (PDF 1715 KB) [file 10142_2023_1050_MOESM1_ESM.pdf]

Supplementary Figure legends

Figure S1. A. Diagram to show collection sites of proximal and distal lung samples from WT and *CFTR*$^{-/-}$ (CF) animals. B. Diagram to show sheep development time course and sample collection points.

Figure S2. Principal component analysis plots showing RNA-seq sample distribution of WT and *CFTR*$^{-/-}$ (CF) animals. A, Proximal lung; B, Distal lung.

Figure S3. Gene ontology process enrichment analyses of differentially expressed genes between gestational time points in WT sheep proximal lung. Differentially expressed genes were filtered to enrich for genes with a fold change ≥ ±1.5 and adjusted p-value ≤ 0.001. Gene ontology analysis by DAVID and only the top 20 biological processes (BP) are shown. A, BP from genes upregulated at 100- compared to 80 days; B, BP from genes downregulated at 120- compared to 100 days; C, BP from genes upregulated at term compared to 120 days; D, BP from genes downregulated at term compared to 120 days.

Figure S4. Gene ontology process enrichment analyses of differentially expressed genes between gestational time points in WT sheep distal lung. Analysis methods as described in Fig. S3. Gene ontology analysis by DAVID and only the top 20 biological processes (BP) are shown. BP from genes upregulated at term compared to 120 days.

Figure S5. Gene ontology process enrichment analyses of differentially expressed genes between gestational time points in *CFTR*$^{-/-}$ sheep distal lung. Analysis methods as described in Fig. S3. A, BP from genes upregulated at term compared to 120 days by DAVID; B, BP from genes downregulated at term compared to 120 days by DAVID.

Figure S6. Developmental profiles of *SLC26A9* in WT and *CFTR*$^{-/-}$ sheep proximal and distal lung. Data on the left are expression profiles in proximal lung and on the right in distal lung. Normalized read counts from RNA-seq data are shown. WT samples are black dots and *CFTR*$^{-/-}$ (CF) samples are grey squares. Values from 2 biological replicates at each time point are shown.

A

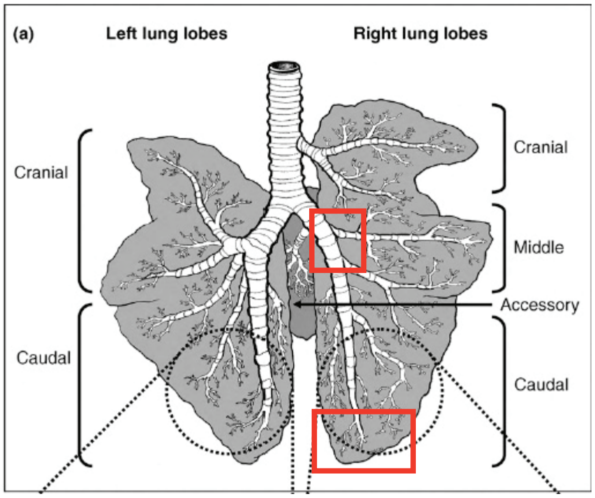

• Image from 10.1016/j.ddmod.2009.12.002

B

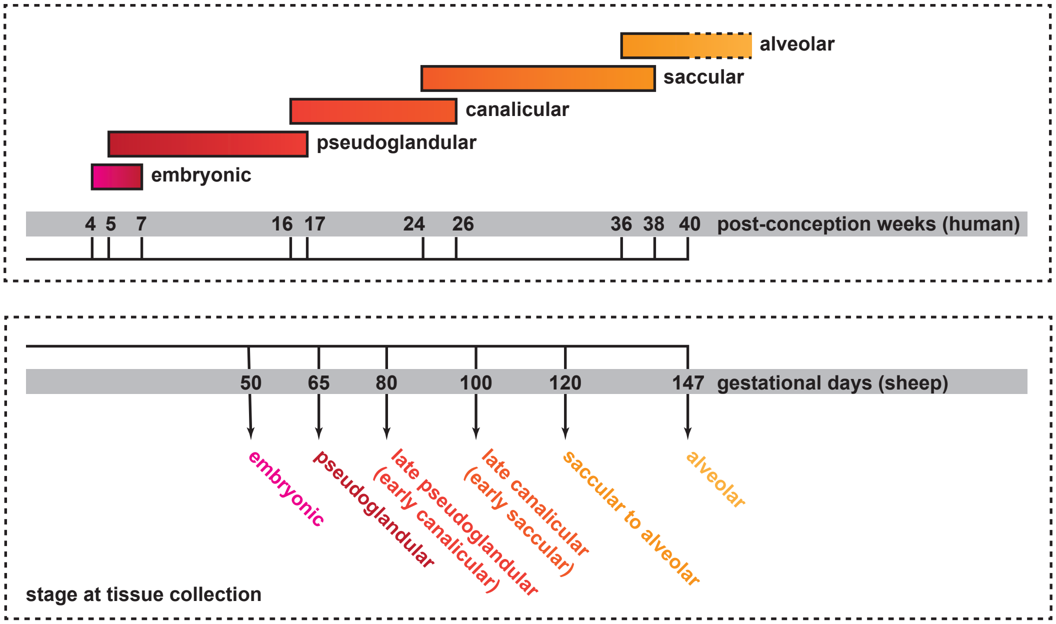

Figure S1

A. Proximal and distal lung tissue collection sites shown as upper and lower red boxes, respectively.
B. Diagram to show lung developmental stages and tissue collection timepoints.

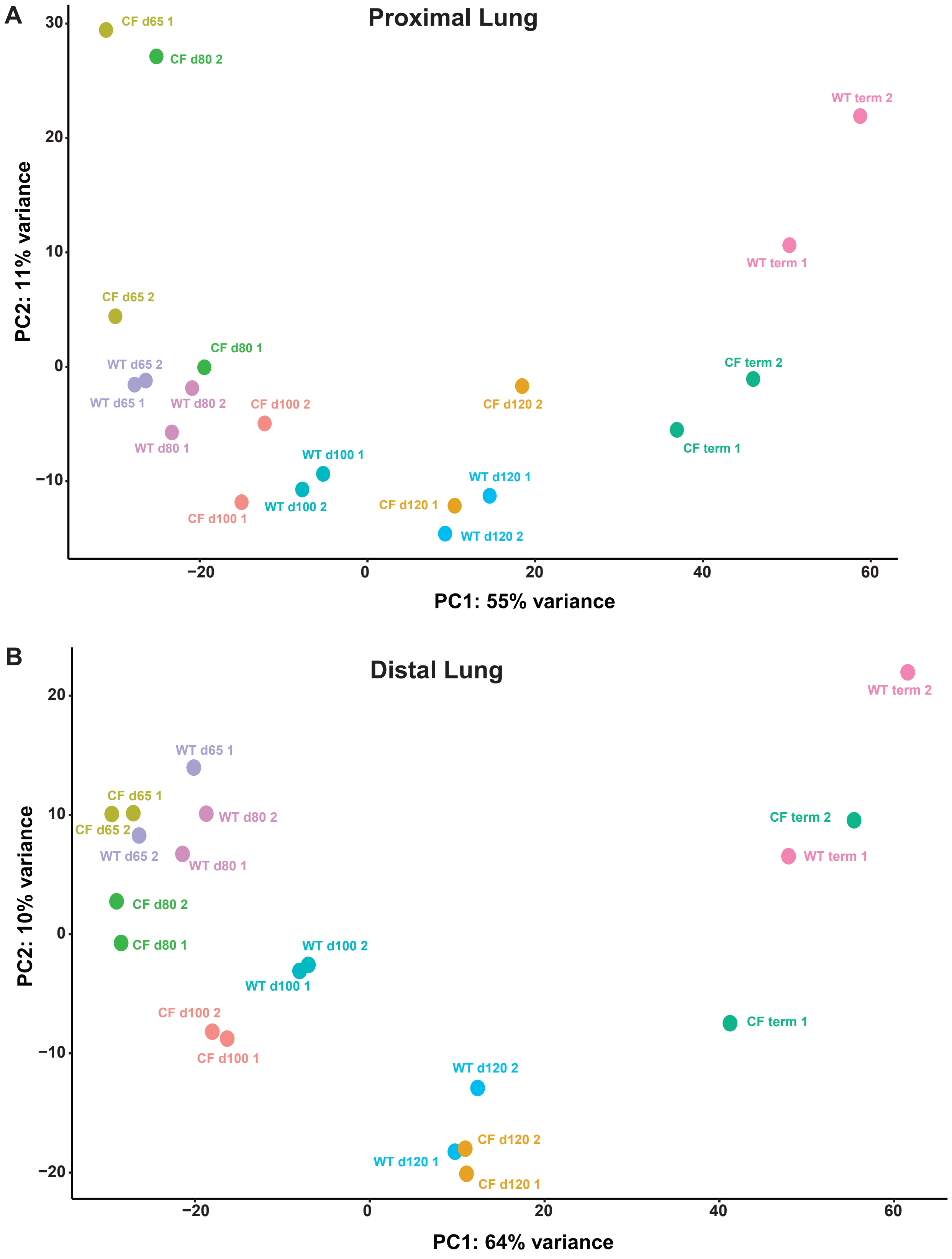
A
Proximal Lung
PC2: 11% variance
PC1: 55% variance
30
20
10
0
−10
−20
0
20
40
60
CF d65 1
CF d80 2
WT term 2
WT term 1
CF d65 2
CF d80 1
WT d65 2
WT d65 1
WT d80 2
CF d100 2
CF d120 2
CF term 2
CF term 1
WT d80 1
WT d100 1
WT d120 1
CF d100 1
WT d100 2
CF d120 1
WT d120 2
B
Distal Lung
PC2: 10% variance
PC1: 64% variance
20
10
0
−10
−20
WT term 2
WT d65 1
CF d65 1
CF d65 2
WT d80 2
CF term 2
WT d65 2
WT d80 1
WT term 1
CF d80 2
CF d80 1
WT d100 2
WT d100 1
CF d100 2
CF d100 1
CF term 1
WT d120 2
CF d120 2
WT d120 1
CF d120 1

Figure S2

Wildtype Proximal Lung

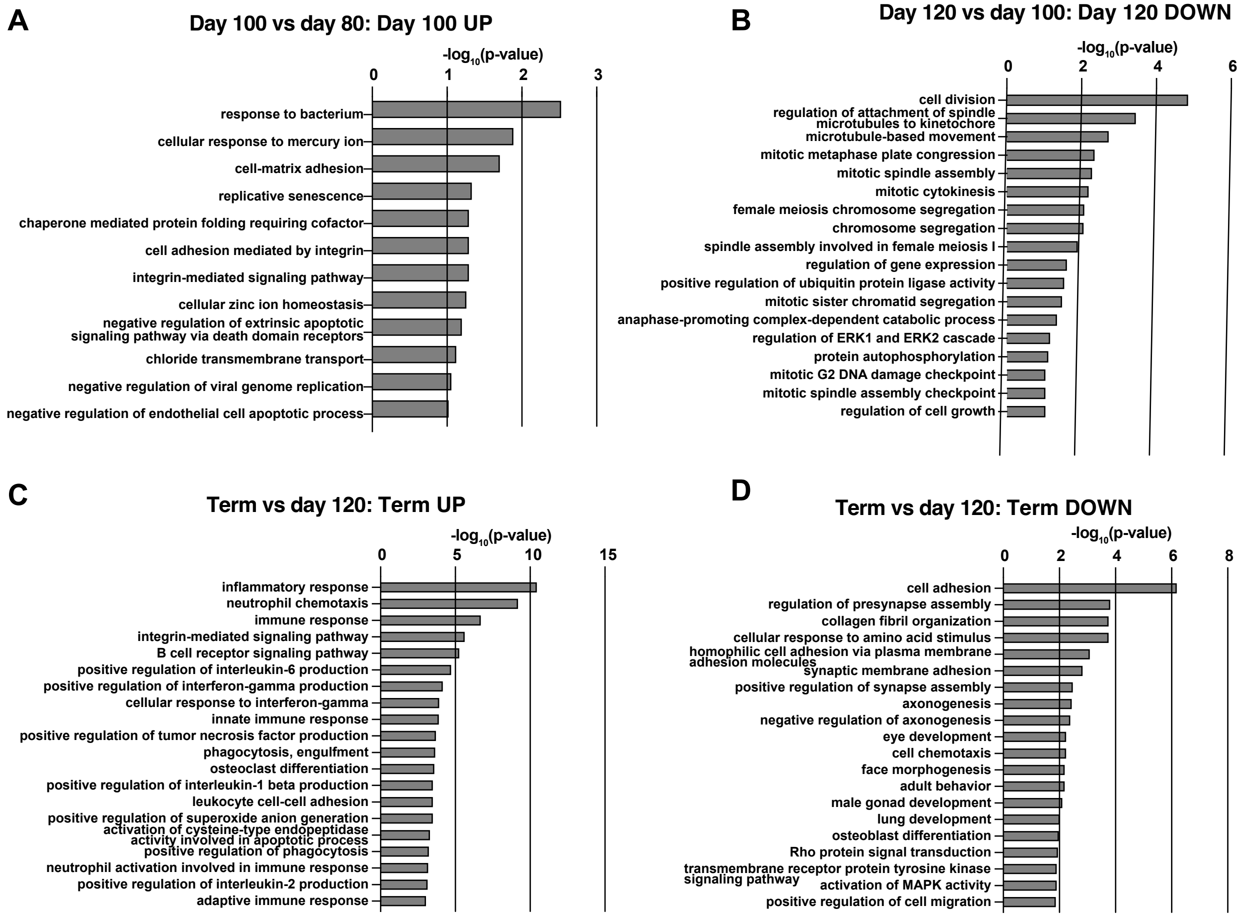

DAVID

Figure S3

Wildtype Distal Lung

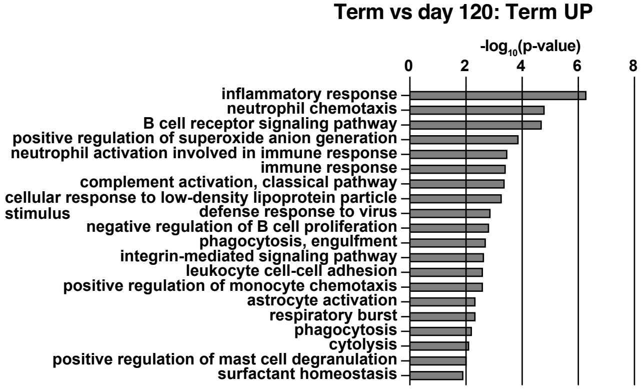

Figure S4

DAVID

CFTR$^{-/-}$ Distal Lung

A

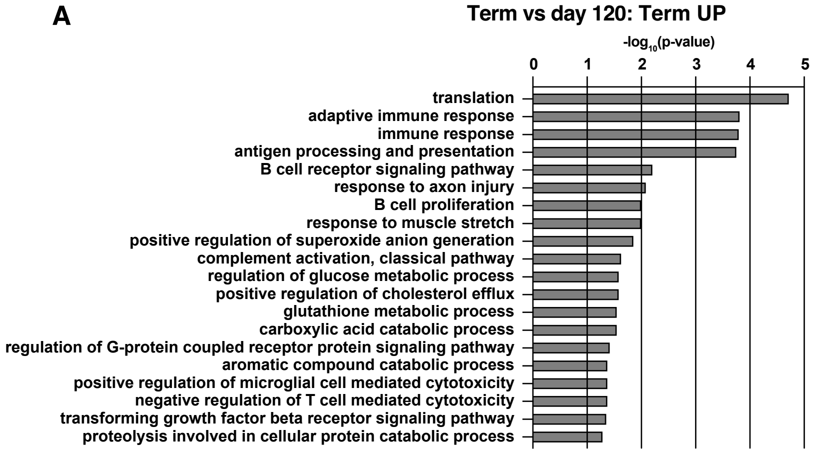

B

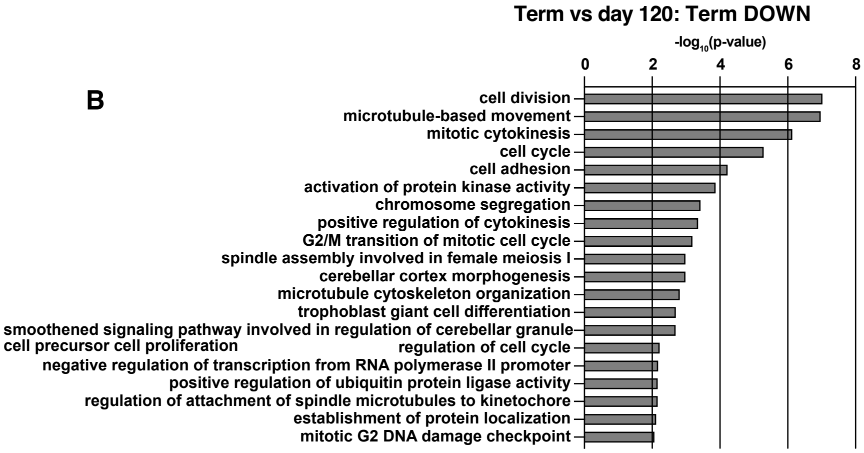

DAVID

Figure S5

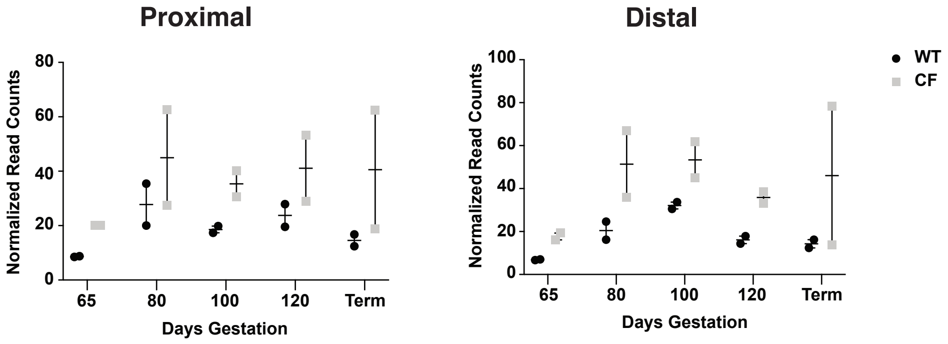

SLC26A9

Figure S6
